# Diagnostic Laboratory Tests for COVID-19 in US: Methodology and Performance

**DOI:** 10.21203/rs.3.rs-43374/v1

**Published:** 2020-07-20

**Authors:** Mrigendra M. Bastola, Craig Locatis, Paul Fontelo

**Affiliations:** NIH/NLM/LHC

**Keywords:** COVID-19, EUA, Approved Lab Tests

## Abstract

**Background:**

COVID-19 is a global pandemic caused by a new coronavirus strain. Innovative tests have been developed to diagnose and characterize the spread of COVID-19. Only a few studies have reported the diagnostic value of currently available tests. The diagnostic performance of the tests is a major concern after the recent resurgence in COVID-19.

**Methods:**

Published papers and FDA data on the currently available tests were used for analysis. Likelihood ratios, and predictive values of tests were computed. Only FDA approved tests were included. RT-PCR performance among different specimen types were also explored.

**Main results:**

All the published reports on the COVID-19 tests reported RT-PCR as the validation tool for their results. Not all available COVID-19 tests reported their sensitivity and specificity. Among the publications which reported, the positive likelihood ratio ranged between 0.15 to 0.88 and tests had high negative likelihood ratio (0.99).

**Conclusion:**

Although most recent publications showed high positive and negative likelihood ratios and high predictive values, the publications on test accuracy and validity have limited scope primarily due to their small sample size and insufficiencies in methodology and published data. Although most lab tests reported high sensitivity and specificity, false omission and false discovery rates were found notable in several COVID-19 lab tests. These results suggest need for caution on test results’ interpretation. Practitioners also need to integrate evidence that is evolving rapidly.

## Background

The virus SARS-CoV-2, which causes the illness COVID-19 was first detected in late 2019. To date (July 1, 2020), more than 10 million COVID-19 cases have been reported with more than half a million deaths worldwide, and the numbers are expected to increase [1]. The rapid detection of COVID-19 cases in the United States will assist in case diagnosis, contact tracing, and help mitigate further spread. A public health emergency determination was made on February 4, 2020 authorizing of emergency use of invitro diagnostic tests for the diagnosis of COVID-19 [2]. As allowed by the FDA, 7 states in USA permit certified laboratories to develop and perform COVID-19 tests without filing an Emergency Use Authorization (EUA) [3]. When validated, the FDA allows the development and distribution by commercial manufacturers, or development and use by laboratories of serology tests to identify antibodies to SARS-CoV-2 [3]. As of May 29, 2020, there were 63 test kit manufacturers and commercial laboratories offering COVID-19 testing listed on the FDA website [2]. 51 were molecular tests and 12 serology tests. A few companies had developed both. All tests that were listed were certified for use only in CLIA (Clinical Laboratory Improvement Amendments) to perform high complexity tests, 21 in the moderately complex test category and only 4 were deemed eligible for use in patient care settings [1].

Several of these laboratory tests are currently allowed under EUA, and state governments can perform tests in certified laboratories and physician provider offices. The test reports from these non-EUA tests must indicate that the test has not been FDA reviewed, a negative test does not rule out COVID-19, the test report must be used with clinical features for diagnosis of COVID-19 and the test reports might be due to present or past infection with SARS-CoV-2 coronavirus strains [3]. Although this information cautions users, it provides little help in understanding the performance of the available COVID-19 tests. The performance of these available tests is a major concern after the recent resurgence in COVID-19 cases.

This brief report of published articles and FDA data explores the methodology, accuracy, sensitivity, and likelihood ratios and predictive values of positive and negative tests of these FDA approved tests and EUA tests. The review provides insights into false negatives, low sensitivities, predictive values, methodology and questionable performance of currently most used COVID-19 tests and specimens, which are relevant in the context of the rising pandemic.

## Methods

A PubMed search for journal publications on the topic, “laboratory diagnosis of COVID-19” completed on April 19, 2020, returned 319 articles. This report only included journal articles that addressed laboratory diagnosis and excluded publications that discussed diagnosis based on clinical features and imaging studies. Only laboratory tests were reviewed and included most used and FDA recognized molecular-bases tests, serological tests, ELISA based tests and chemiluminescence tests. The positive and negative likelihood ratios, and positive and negative predictive values of tests were calculated based on the current national incidence of 7542.61/ per million (national average as of July 1, 2020, see also Table 1& 2). The prevalence needed in the calculation process was replaced by the incidence rates because the COVID-19 has same prevalence and incidence rates for 2020, and there were no prior cases in US before January 2020[1]. All tests were either FDA approved, or currently in the approval process. Tests that had not provided their data on sensitivity and specificity were excluded in this review (As of July 1, 2020). Additionally, tests approved for research surveillance purposes only were excluded in this study. Because most of the publications reported only sensitivity and specificity and did not report either true or false data on positive and negative tests, this report calculated predictive values directly from sensitivity and specificity values. Final selection included seven papers and online publications, which sufficiently reported relevant data on test performance and test methodology (Table 1& 2).

## Results

Included studies, data collected, and results are shown in Table 1 & 2. All the tests used RT-PCR for validation. Only two studies directly compared the accuracy rates for nasopharyngeal versus oropharyngeal swabs, and both had insufficiencies in sample size, deficiencies in reporting false/true values for positive and negative tests, and description of methods [4].

The sensitivity of the RDTs varied between 87 to 97% while the specificity varied between 96 to 100%. Among the publications which reported, the positive likelihood ratio ranged between 0.15 to 0.88 and tests had high negative likelihood ratio (0.99). Several tests had high false detection rate, and some had high false omission rates (Table 1). Of the samples collected for RT-PCR, bronchoalveolar lavage had the highest sensitivity (93%) and specificity (100%), while nasopharyngeal and sputum had higher sensitivities compared to oropharyngeal swabs (Table 2).

## Discussion

Although sensitivity and specificity of the tests have not been well established, extensive testing has been advocated for early diagnosis and quarantine of the COVID-19 patients to help mitigate the spread of the disease, as practiced in South Korea [5]. Nevertheless, the published data on sensitivity and specificity of the commonly used tests are neither consistent nor clear, with not enough published data on false positives and false negatives [6]. Apart from lymphocytopenia (in 83% of COVID-19 positives), the rest of the routine blood work was inconsistent and non-specific [7]. Currently, vaccines are still undergoing clinical trials [8]. Levels of neutralizing antibodies needed, and protective herd immunity remains uncertain in COVID-19. Published reports suggest that herd immunity may develop only after extensive vaccination or after at least 68% of the population recover from COVID-19 [9]. Although the FDA has recently approved home based test kits for COVID-19, their performance is still unknown [10, 11, 12].

This brief report summarizes published results of currently approved COVID-19 tests has several implications. The positive and negative likelihood ratios mirror the sensitivities and specificities because most of the studies exclusively reported only true positive and true negative results (Table 1&2) [13]. A caveat with molecular testing is that the viral RNA might be undetectable within a week of infection and antibodies IgG and IgM rise after day 9 onwards [14]. Tests which have high sensitivity are best used for screening symptomatic patients, while tests with highest specificity are best for confirming diagnosis, so patients with positive tests can treated or quarantined [15]. Both false negative and false positive tests may lead to erroneous decisions in managing patients, such as contact tracing, isolation, and treatment [6,16]. Additionally, false negatives may have greater importance among health care workers who might otherwise appear to be asymptomatic. Moreover, finding high SARS-CoV-2 RNA in feces that was not previously reported, has raised public health concerns [9]. There is limited published data on tests that are approved outside the US. Comparable results are seen on the results of this study to these limited data on tests approved outside USA reported 87 to 100 percent sensitivity and reported range from 98.7 to 100% specificity [11].

Tests having high sensitivity providing quick results in physicians’ offices or in clinic settings can be valuable additional tools in tracking COVID-19 patients and asymptomatic individuals and can help provide an epidemiologic profile of a community. Since no single test can attain 100% sensitivity and 100% specificity, the best approach to testing in COVID-19 would probably come from a combination of tests with high sensitivity in the first and high specificity in the second [17]. Cross-reactivity with other types of non-virulent coronavirus has not been established for RDTs, hence the caution is needed in reporting results. The value of the antibody-based tests on diagnosing individuals with COVID-19 in recovery or past infection with COVID-19 is still not fully understood. Furthermore, long-term immunity after COVID-19 infection is still uncertain, which could undermine the effectiveness of a future vaccine if no long-term or strain-specific neutralizing antibodies are produced in adequate quantities.

Published results showed that several sample types for RT-PCR based tests had low sensitivity and significant false omission and false discovery rates (Table 1 and Table 2). These have been explained by specimens collected too early when viremia was low or after patients presented clinically with signs and symptoms and may have passed the active viremia phase, or due to technical issues. Only a few studies support the use of saliva and nasopharyngeal samples for testing. Sputum specimens and nasopharyngeal swabs yielded 69% and 63% sensitivity, respectively, suggesting that sputum specimens might have adequate viral nucleic acids for detection using the RT-PCR assay [18]. Moreover, salivary viral load was highest during the first week after symptom onset but subsequently declined over time [18]. In a large study conducted in China, the positive rate was 57% for selected individuals presenting with fever in clinics at the epicenter of the epidemic [19]. Older age was correlated with higher viral load [19]. In another study, the presence of antibodies and seropositivity rates were higher for anti-RBD (receptor binding domain) IgG and anti-NP (nucleoprotein) IgG, than anti-RBD IgM and anti-NP IgM [20]. Viral RNA has been reported 37 days after symptom onset [21]. Whether patients are still infective at this point is unknown.

The results of the ongoing pandemic in this review indicate that the best sensitivity is attained using bronchoalveolar fluid (93%) followed by saliva (92%), sputum (69%) then nasopharyngeal swabs (63%) and serum (41%) (Table 2). Feces only yielded 29% positivity. Perplexing is the low sensitivity of blood samples (1%). Specimen collection and processing and specimen preservation and transport could have factored significantly into the results found.

The findings of this review paper support the emphasis on COVID-19 testing made by WHO, however, even the level of diagnostic accuracy might not be sufficient for effective control of the pandemic [22]. A recent study which analyzed samples from known COVID-19 individuals, percent seropositive increased with time interval, peaking at 81.8–100.0% in samples taken >20 days after symptom onset [23]. A few laboratories were developing molecular-based tests that were authorized for use in their laboratory only. These are laboratories that perform high complexity tests authorized through EUAs. Some tests were developed outside the US and have been purchased by some states or imported by companies in the US. These tests may not have been authorized by the FDA or are in the process of applying for EUAs. As testing methods are continuously evolving, newer tests may attain higher specificities and sensitivities, either alone or in combination.

### Limitations:

Limited published data on performance of the lab tests for COVID-19, including data on true and false positive rates on positive and negative tests is a major limitation of the study, which led authors to rely on direct computation of predictive values from reported sensitivity and specificity values. The results in this review must be interpreted in the context of exposure history and other supporting evidence. Multiple factors besides test performance and false omission rates discussed in review are possibly aiding undetected COVID-19 cases. Although not necessarily a limitation in this review, geographic locations could have introduced their own unique demographic characteristics in the studies. As in most laboratory tests, the predictive value, both positive and negative, depend greatly on the prevalence of disease in the population. This study also assumes that the sample collection and processing methods were comparable in all included studies. This review included only a select number of articles, and could not have included all publications, which are added daily and at a rapid pace. This study used the most recent published prevalence rate from CDC in the USA, which is subject to change if the pandemic progresses, and higher prevalence gives higher performance for the COVID-19 tests. In Tests approved in countries other than US were excluded in this study due to limited published data on their performance.

## Conclusion

Most of the recently published studies of tests on COVID-19 are limited in applicability primarily because of their small sample size, insufficient methods, and available data in publications. Although most lab tests reported high sensitivity and specificity, false omission and false discovery rates were found notable in several COVID-19 lab tests. However, there are suggestions of which specimen sources might yield the best sensitivity and specificity. The results must be interpreted with caution and practitioners need to integrate the most recent evidence that is evolving rapidly. A careful selection of tests with high sensitivity and specificity would be highly beneficial in specimen selection and in efforts to mitigate the spread of COVID-19.

## Figures and Tables

**Table 1:** Summary of the relevant data reported in included studies on FDA approved COVID-19 lab tests on Blood, Serum or Plasma specimens[11, 28].

FDA Approved COVID-19 Tests	Sensitivity	Specificity	Positive LR	Negative LR	Positive PV*	Negative PV*	False Omission Rate	False Discovery Rate
***Enzyme Linked Immunosorbent Assay***								
• ELISA (VITROS)	83	99.9	83	0.17	0.863	0.998	0.0013	0.1368
• ELISA (Mt. Sinai)	NA	NA						
• ELISA (Ortho-Clinical)	88	99.9	88	0.12	0.869	0.999	0.0009	0.1300
• ELISA (Diasorin)	94	98	4.7	0.065	0.263	0.999	0.0005	0.7368
• ELISA (Bio-Rad)	98	99	9.8	0.020	0.426	0.999	0.0002	0.5731
• ELISA (EuroImmmun))	99.9	99.9	99.9	0.001	0.883	0.999	<0.0001	0.1163
• ELISA (InBios)	97.8	98.9	8.89	0.022	0.403	0.999	0.0002	0.5967
• ELISA (Emory University)	99.9	97.7	4.34	0.001	0.248	0.999	<0.0001	0.7518
***Rapid Diagnostic Assays for IgM & IgG***								
• RDT (Cellex)	94	96	2.35	0.0625	0.151	0.999	0.0005	0.8484
• RDT (ChemBio)	NA	NA						
• RDT (Aytu)	97	99.9	97	0.030	0.88	0.999	0.0002	0.1194
• RDT (Innovita)	87	99.9	87	0.130	0.868	0.999	0.001	0.1313
• RDT (Advaite)	89	99.9	89	0.110	0.871	0.999	0.0008	0.1288
• RDT (Autobio & Hardy)	97	99	9.7	0.030	0.424	0.999	0.0002	0.5756
• RDT (Healgen Scientific)	96.7	97	3.22	0.034	0.196	0.999	0.0003	0.8032
• RDT (Hangzhou Biotest)	91.56	99.52	19.08	0.085	0.591	0.999	0.0006	0.4082
• RDT (Siemens Healthcare)	99.9	99.8	49.95	0.001	0.791	0.999	<0.0001	0.2084
• RDT (Biohit Healthcare)	56.6	99.46	10.48	0.436	0.443	0.996	0.0033	0.5566
• RDT (Hangzhou Laihe)	85.7	99.43	15.03	0.144	0.533	0.998	0.0011	0.4667
***Chemiluminescent Immunoassay***								
• CLIA (Roche)	99.9	99.8	49.95	0.001	0.791	0.999	<0.0001	0.2084
• CLIA (Wadsworth & NYSDH)	99.9	99.9	99.9	0.001	0.883	0.999	<0.0001	0.1163
• CLIA (Vibrant America)	98.1	98.6	7.01	0.019	0.347	0.999	0.0001	0.6525
• CLIA (Siemens)	97.3	99.8	48.65	0.027	0.787	0.999	0.0002	0.2128
• CLIA (Babson)	66.7	99.9	66.7	0.333	0.835	0.997	0.0025	0.1647
• CLIA (Beckman Coulter)	95.3	99.9	95.3	0.047	0.878	0.999	0.0004	0.1213
***Quantification of Interleukin-6 levels***								
• CLIA (Roche)	84	63	0.23	0.254	0.016	0.998	0.0019	0.9830

* Calculations of predictive values are based on Incidence of COVID-19 in USA (National average, 1^st^ July 2020)^[24]^

# values after 21 days onwards.

LR=Likelihood Ratio. PV=Predictive Value. NA=No Data Available.

**Table 2:** Summary of RT-PCR test performance in different specimen types.

Methods	N	Sensitivity	Specificity^#^	Positive LR	Negative LR	Positive PV*	Negative PV*	False Omission Rate	False Discovery Rate
**Commonly used Samples (RT-PCR)**									
• Throat Swabs PCR^[25]^	1070	59	100	59.58	0.41	0.817	0.996	0.0031	0.1823
• Oropharyngeal swabs^[4]^	205	48	100	44.072	0.52	0.784	0.996	0.0039	0.2151
• Nasopharyngeal swabs^[4]^	490	63	100	57.845	0.37	0.827	0.997	0.0028	0.1727
• Sputum^[9]^	104	69	100	63.354	0.31	0.839	0.997	0.0023	0.1601
**Other less commonly used Samples (RT-PCR)**									
• Bronchoalveolar fluid^[9]^	14	93	100	85.390	0.07	0.876	0.999	0.0005	0.1239
• Saliva^[26]^	12	92	100	84.472	0.08	0.874	0.999	0.0006	0.1251
• Feces^[9]^	153	29	100	26.627	0.71	0.687	0.994	0.0053	0.3121
• Blood^[9]^	307	1	100	0.918	0.99	0.070	0.992	0.0074	0.9293
• Serum^[27]^	95	41	100	37.645	0.59	0.757	0.995	0.0044	0.2429

* Calculations of predictive values are based on Prevalence of COVID-19 in USA (National average, 1^st^ July 2020)^[24]^.

# Assuming PCR specificity for COVID-19 100% across all specimen types.

LR=Likelihood Ratio. PV=Predictive Value.

## Abbreviations

COVID-19: Coronavirus Diseases 2019
CLIA: Clinical Laboratory Improvement Amendments
ELISA: Enzyme-linked immunosorbent assay
EUA: Emergency Use Authorizations
FDA: Food and Drug Administration
Ig G: Immunoglobulin G
Ig M: Immunoglobulin M
LR: Likelihood Ratio
NP: Nucleoprotein
NYSDH: New York State Department of Health
PCR: Polymerase Chain Reaction
PV: Predictive Value
RBD: Receptor Binding Domain
RDT: Rapid Diagnostic Test
RT-PCR: Reverse Transcription Polymerase Chain Reaction
SARS-CoV-2: Severe Acute Respiratory Syndrome – Coronavirus -2
WHO: World Health Organization
